# Tailoring of Geranium Oil-Based Nanoemulsion Loaded with Pravastatin as a Nanoplatform for Wound Healing

**DOI:** 10.3390/polym14091912

**Published:** 2022-05-07

**Authors:** Waleed Y. Rizg, Khaled M. Hosny, Bayan A. Eshmawi, Abdulmohsin J. Alamoudi, Awaji Y. Safhi, Samar S. A. Murshid, Fahad Y. Sabei, Adel Al Fatease

**Affiliations:** 1 Department of Pharmaceutics, Faculty of Pharmacy, King Abdulaziz University, Jeddah 21589, Saudi Arabia; wrizq@kau.edu.sa (W.Y.R.); beshmawi@kau.edu.sa (B.A.E.); 2 Center of Excellence for Drug Research and Pharmaceutical Industries, King Abdulaziz University, Jeddah 21589, Saudi Arabia; 3 Department of Pharmacology and Toxicology, Faculty of Pharmacy, King Abdulaziz University, Jeddah 21589, Saudi Arabia; ajmalamoudi@kau.edu.sa; 4 Department of Pharmaceutics, Faculty of Pharmacy, Jazan University, Jazan 82817, Saudi Arabia; asafhi@jazanu.edu.sa (A.Y.S.); fsabei@jazanu.edu.sa (F.Y.S.); 5 Department of Natural Products and Alternative Medicine, Faculty of Pharmacy, King Abdulaziz University, Jeddah 21589, Saudi Arabia; samurshid@kau.edu.sa; 6 Department of Pharmaceutics, College of Pharmacy, King Khalid University, Abha 62529, Saudi Arabia; afatease@kku.edu.sa

**Keywords:** nanotechnology, burn wound, ex-vivo permeation, essential oil, statins, Box–Behnken design

## Abstract

The healing of a burn wound is a complex process that includes the re-formation of injured tissues and the control of infection to minimize discomfort, scarring, and inconvenience. The current investigation’s objective was to develop and optimize a geranium oil–based self-nanoemulsifying drug delivery system loaded with pravastatin (Gr-PV-NE). The geranium oil and pravastatin were both used due to their valuable anti-inflammatory and antibacterial activities. The Box–Behnken design was chosen for the development and optimization of the Gr-PV-NE. The fabricated formulations were assessed for their droplet size and their effects on the burn wound diameter in experimental animals. Further, the optimal formulation was examined for its wound healing properties, antimicrobial activities, and ex-vivo permeation characteristics. The produced nanoemulsion had a droplet size of 61 to 138 nm. The experimental design affirmed the important synergistic influence of the geranium oil and pravastatin for the healing of burn wounds; it showed enhanced wound closure and improved anti-inflammatory and antimicrobial actions. The optimal formulation led to a 4-fold decrease in the mean burn wound diameter, a 3.81-fold lowering of the interleukin-6 serum level compared to negative control, a 4-fold increase in the inhibition zone against *Staphylococcus aureus* compared to NE with Gr oil, and a 7.6-fold increase in the skin permeation of pravastatin compared to PV dispersion. Therefore, the devised nanoemulsions containing the combination of geranium oil and pravastatin could be considered a fruitful paradigm for the treatment of severe burn wounds.

## 1. Introduction

Skin, the largest organ of the human body, is an important protective organ that can safeguard the body against various dangers such as pathogens, toxins, ultraviolet radiation, and mechanical damage [1]. Skin has three main layers, namely, the epidermis, dermis, and hypodermis, each of which has a unique composition and function that contributes to the overall protection against external environmental factors [2]. Critical burns and wounds are amongst the most prevalent life-threatening dangers. The healing process of open skin includes the restoration of the skin’s normal protective layer following trauma caused by accident or an intentional surgical procedure. This healing process has several integrated and coordinated successive phases, namely, hemostasis, inflammation, proliferation, revascularization, and remodeling [3,4]. A person’s age, sex, level of stress, and pre-existing medical conditions all affect the time needed for complete wound healing [5]. Wounds associated with diabetes are a significant type that result from the stress diabetes causes in the body. The wound healing process is impaired in diabetic patients due to neuropathy, hypoxia, decreased immunity, fibroblastic dysfunction, and impaired angiogenesis [6,7]. Treatment strategies to accelerate the wound healing process were assessed, and many of them showed positive results [8,9,10].

Statin drugs are among the important cholesterol-lowering agents used currently [11]. Besides their powerful cholesterol-lowering activity, they have desirable effects on wound healing in many animal models [12,13]. Treatment of different wound animal models with statins led to an increase in vascular endothelial growth factor (VEGF), an important inducer of angiogenesis and promoter of wound healing [14,15]. Statins also improved epithelialization and enhanced the mechanical strength of the skin, both of which improved the healing of wounds [16]. Pravastatin (PV) is one of the statins; it was patented in 1980 and approved for clinical use in 1989. Like other statins, PV exerts its hypolipidemic action by inhibiting the enzyme HMG-CoA reductase, which is expressed in the liver and plays a pivotal role in cholesterol output [17]. In a recent clinical trial involving patients who received radiation for head and neck cancer, PV was administered in a dosage of 40 mg/day for 12 months. The results showed that PV was an efficient antifibrotic agent, and this finding supports the theory that PV could reverse radio-induced fibrosis and enhance the wound-healing process [18].

Geranium (Gr) oil is an essential oil extracted from the plant *Pelargonium graveolens* that was cultivated originally in South Africa and then distributed in Asia and the Middle East [19]. Gr oil is known traditionally for its calming effects on emotional distresses such as frustration, anger, and anxiety and its ability to lower high blood pressure [20]. Gr oil possesses immune-boosting properties and immune-modulating properties against natural killer cells [21]. Recent studies showed anticancer activity of this essential oil that resulted from two of its major components, citronellol and trans-geraniol [22,23]. The aerial parts of *P. graveolens* have been used for wound healing because they possess antimicrobial activity against *Staphylococcus aureus, Candida glabrata, Bacillus subtilis, Enterococcus faecalis, Candida krusei, Mycobacterium tuberculosis,* and *Mycobacterium intracellulare* [24]. Rose geranium oil has its own market in the cosmetic, perfume, and aromatherapy industries. Its anti-inflammatory and palliative effects on the skin make this essential oil a good candidate for many topical formulations [25,26].

Self-nanoemulsifying drug delivery systems (SNEDDS) are anhydrous mixtures of a low-solubility drug, an oil, a surfactant, and a cosurfactant with a droplet size of less than 100 nm [27]. These nanoemulsions have been used extensively in recent times in dermal applications owing to their nanosized droplets, which enhance the solubility and transdermal permeation of the incorporated drug [28,29,30]. Different low-solubility drugs from different drug classes, such as antiviral drugs, immunomodulators, nonsteroidal anti-inflammatory drugs, and lipid-lowering agents, have been incorporated in nanoemulsion formulations [31,32,33]. Besides the ease of making nanoemulsion formulations, they readily gain acceptance from authorities because they are considered to be drugs that are generally recognized as safe during manufacturing [34].

A design of experiment (DOE) approach was used in the formulation of the varying dosage forms in this study to choose the optimal formulation and test its biological activity. The DOE has been used extensively in nanotechnology formulations to help researchers study the interactions of drug excipients, solve problems during formulation, study the process parameters and how they affect each other, and reduce the number of experiments to reach the optimal formulation [35].

To our knowledge, no studies have been done to investigate the combined effect of statins and Gr oil in treating burn wounds. Therefore, the objective of this study was to introduce a transdermal drug delivery system containing a Gr oil–based nanoemulsion loaded with PV (Gr-PV-NE). The intended choice of a transdermal drug delivery system was made based on its localized action and the droplet size of the nanoemulsion, which could promote skin membrane permeation and avoid presystemic metabolism and efflux mechanisms [36,37].

## 2. Materials and Methods

### 2.1. Materials

PV was acquired as a generous gift from the Saudi Arabian Japanese (SAJA) Pharmaceutical Company Limited (Jeddah, Saudi Arabia). Gr oil was purchased from the Beutysway Commercial Foundation (Jeddah, Saudi Arabia). Tween 80 and Span 80 were purchased from Sigma-Aldrich Co. (St. Louis, MO, USA). Transcutol was a gift from Gattefosse (Saint-Priest, France). High-performance liquid chromatography–grade methanol and acetonitrile were obtained from Merck (Darmstadt, Germany). All other reagents and chemicals were of analytical grade.

### 2.2. Methods

#### 2.2.1. Experimental Design and Optimization of Self-Nanoemulsion Formulations

With reference to this study, the Box–Behnken design (BBD) was pursued to scrutinize the influence of independent variables on dependent ones. Twenty-three formulations were made with the chosen design using Design-Expert Version 13 Software (Stat-Ease, Inc., Minneapolis, MN, USA). The selected statistical design produced various relationships between the independent variables, and they are summarized in Table 1. The three explored factors were the amount of Gr oil in milligrams (A), amount of PV in milligrams (B), and hydrophilic-lipophilic balance (HLB) of the surfactant mixture (Tween80/Span80) (C) in the prepared nanoemulsion. The estimated responses were the globule size of the prepared NEs (Y_1_) and the mean burn wound diameter (Y_2_). Preliminary studies were followed to select the factors’ levels.

#### 2.2.2. Gr-PV-NEs Preparation

The production procedure had two steps. The first step was the fabrication of the plain SNEDDS, in which a Gr oil concentration of 10%, 20%, or 30% (according to the design) was blended with 60% of the surfactant mixture with an HLB of 11, 12, or 13 (according to the design) and then brought to 100% by the Transcutol cosurfactant. In the second step, PV was mixed with the plain NEs with the aid of sonication in concentrations of 10, 20, or 40 mg/g of the prepared self-nanoemulsion according to the design, as shown in Table 2.

#### 2.2.3. Determination of Globule Size

The droplet size and polydispersity index (PDI) of each of the fabricated Gr-PV-NEs was examined by diluting 100 μL of each formulation with 900 μL of double distilled water in a volumetric flask. Next, the diluted samples were vigorously mixed, and 100 μL of the dispersed sample was withdrawn to determine its droplet size; this was done with a Zeta track particle size analyzer (Microtrac, Inc., Montgomeryville, PA, USA) [38]. Assessment of the sample was carried out in triplicate, and the results were presented as the mean ± standard deviation (SD). PDI is a good tool for evaluating foemulation homogeneity.

#### 2.2.4. Assessment of Wound Healing

##### Animal Handling and Care

The guidelines of the Animal Ethics Committee, Beni Suef Center for Laboratory Animals, Beni Suef, Egypt, were adopted in animal handling and care. Investigators abided by the guidelines articulated in the Declaration of Helsinki and its Guiding Principles in the Care and Use of Animals (NIH Publication No. 85-23, 1985 revision) and adopted the ethical approval of the protocol before experimentation (Approval No. 26/4-21).

The experimental rats were obtained and kept in laboratory cages with free access to food and water. Animals’ suffering was minimized according to the guidelines. The animals were kept for a minimum of 14 days prior to the investigation under standard conditions of temperature (25 ± 1 °C) and relative humidity (55 ± 5%) with a 12 h light and 12 h dark cycle.

Sixty-nine rats were used in the study; they were divided into 23 groups, with 3 rats per group. Each group was treated with one of the formulations developed by the experimental design. An intraperitoneal dose of thiopental of 15 mg/kg was administered simultaneously with an intramuscular dose of 25 mg/kg of ketamine. The hair on the rat’s back was shaved, and the skin was sterilized with an alcohol swab. Following that, burn wounds were induced on the back skin using 1.5 cm skin biopsy heated bunches. Then, the investigated formulation for each group was applied once daily for a period of 14 days [39].

##### Measurement of Burn Wound Diameter

After the application of each formulation once a day for 14 days, a caliper was used to measure the average diameter of the induced wound lesions in each group to evaluate the second response for each formulation. Measurements were done in triplicate, and the findings were introduced as the mean ± SD.

### 2.3. Optimization of the Gr-PV-NEs

The desirability function was the basis of the Design-Expert software for obtaining the optimal NE formulation. The main objective of the optimization process was to find a formulation with the smallest droplet size and mean burn wound diameter. The software chose the solution with the desirability value closest to 1. To guarantee the model’s validity and adequacy, the elected formulation was fabricated, depicted, and eventually compared with the response values expected by the software.

### 2.4. Characterization of the Optimized Formulation

#### 2.4.1. Determination of Entrapemnt Efficiency

Percentage drug entrapment efficiency (EE%) was determined for the optimum Gr-PV-NE formulation (containing 40 mg PV) using an indirect method. Sample was centrifuged at 15,000 rpm for 30 min using cooling centrifuge (SIGMA 3–30K, Steinheim Germany). After centrifuge, 1 mL of supernatant transparent layer was diluted with 10 mL distilled water. The PV amount in the sample was determined by a reported high-performance liquid chromatography (HPLC) method. In short, the withdrawn samples were diluted with the mobile phase, which composed of 10 mM ammonium acetate, methanol, and triethylamine in a ratio of 40:60:0.17 *v/v/v*. Ten microliters of the prepared samples were injected with a flow rate of 1.0 mL min^−1^. The PV detection wavelength and elution time were set to be 239 nm and 2.15 min, respectively. Results were taken in triplicate and the average was taken into consideration and EE% was calculated using the following equation.

EE% = [TD−FD]/TD × 100(1)

where TD is the total added drug amount, FD is the free unentrapped drug

#### 2.4.2. Zeta Potential Determination

Zeta potentiap (ZP) of optimum Gr-PV-NEs (containing 40 mg PV) was examined via diluting 100 μL of the formulation with 900 μL of double distilled water in a volumetric flask. The diluted samples were thoroughly mixed, and 100 μL of the mixed samples were employed to determine its ZP using a Zeta track analyzer (Microtrac, Inc., Montgomeryville, PA, USA) [38]. Assessment of the sample was carried out in triplicates, and the results were presented as the mean ± standard deviation (SD).

#### 2.4.3. Measurement of Burn Wound Diameter and Interlukin-6 Level

As in previously described evaluations of different Gr-PV-NE formulations, the test in this study was repeated on 15 rats that were divided into 5 groups. The first group (Group A) was treated with the optimum formulation proposed by the design and followed to determine the outcomes of the first stage of the animal test. The second group (Group B) was treated with the optimum formulation that contained no PV. The third group (Group C) was treated with the optimum formulation prepared utilizing oleic acid in lieu of Gr oil. The fourth group (Group D) was treated with Gr-PV mixture. The fifth group (Group E) was treated with normal saline and served as a negative control. The test continued for 14 days. The formulations were applied once a day and the required parameters were evaluated. All tested formulations contained PV amount equal to 40 mg/g of the tested formulation.

For the assessment of interleukin-6 (IL-6), a previously described quantitative sandwich enzyme-linked immunoassay technique (R&D Systems, Inc., Minneapolis, MN, USA) was employed. A microplate was covered with a particular monoclonal antibody that was specified for rat IL-6. Consequently, the animals’ IL-6 that was present in the tested sample was engaged by the antibody. Following the elimination of any unbound interleukin, an enzyme-linked polyclonal antibody particular for rat IL-6 was appended, and the original blue color was transformed into a yellow one. Finally, the IL-6 level in the tested samples was evaluated by measuring the intensity of the produced color [36].

#### 2.4.4. Ex-Vivo Permeation Study

The test was performed according to a previously described procedure [40]. A freshly excised section of abdominal rat skin was obtained from a male Wistar rat and utilized to determine the permeation profile of PV from various formulations (i.e., Gr-PV-NE optimum formulation, PV-NE prepared with oleic acid instead of Gr oil, Gr-NE prepared without PV, physical mixture of PV and Gr oil, PV aqueous dispersion, and plain NE prepared without PV or Gr oil). All formulations except for the plain nanoemulsion contained PV amount equal to 40 mg/g of the tested formulation. The institutional animal ethics committee approved the experimental protocol. In brief, the abdominal skin hair was guardedly clipped and removed without causing any skin damage. Then, rats were slaughtered, and abdominal skin was separated from the subsidiary connective tissues and cleaned with a Ringer solution before experimentation. Automated Franz diffusion cells (MicroettePlus; Hanson Research, Chatsworth, CA, USA) having a permeation area of 1.76 cm^2^ were employed for determining the ex-vivo skin permeation of the PV from the previously stated formulations. The skin (2.5 × 2.5 cm^2^) was inserted between the donor and receptor chambers of the cells with the stratum corneum layer oriented toward the donor compartment. The receptor milieu was phosphate buffer saline (PBS, pH 7.4) adjusted at 37 ± 0.5 °C in a sufficient volume to meet the sink condition for ex-vivo permeation studies. One milliliter of the various tested formulations was applied to the stratum corneum layer of the skin through the donor chamber and covered with Parafilm to diminish evaporation. At time intervals of 1, 2, 4, 6, 8, 10, 12, and 24 h, 0.5 mL aliquots were extracted from the receptor compartment. Immediately, a fresh receptor milieu previously heated to 37 ± 0.5 °C replaced the withdrawn samples. The PV amount in the gathered samples was determined by a reported high-performance liquid chromatography (HPLC) method previously described in Section 2.4.1.

#### 2.4.5. Antibacterial Activity Evaluation

The antimicrobial action of the previously stated formulations (Section 2.4.2) was examined applying a disc diffusion method [41]. The well-known Gram-positive bacterium *S. aureus*, ordinarily present in infected burns, was utilized as a test bacterium. Following the procedure endorsed by the Clinical and Laboratory Standards Institute, an *S. aureus* suspension was made to the 0.5 McFarland turbidity standard and mounted on Mueller–Hinton agar plates. Filter-paper discs (with a diameter of 10 mm) were immersed in the tested samples and then put in the center of the agar plate and incubated for 24 h at 37 °C. When inhibitory concentrations were reached, a clear so-called inhibition zone containing no colonies could be seen around the discs. Lastly, the inhibition zones’ diameters were measured for each examined formulation.

#### 2.4.6. Statistical Analysis

Compiled IL-6 serum level data were tested using the one-way ANOVA followed by the post-hoc Tukey honest significant difference (HSD) test for multiple comparisons, and the level of significance was set at a *p*-value of less than 0.05 using SPSS software (version 22, Chicago, IL, USA). The obtained data were tested for normality using the Kolmogorov–Smirnov (K-S) test.

## 3. Results and Discussion

### 3.1. Assessment of Gr-PV-NE Droplet Size

Regarding the topical administration of active agents, the physicochemical specifications of nanoemulsions are fundamental factors that must be determined in the fabrication process [42].

The droplet size is an essential key parameter and can identify an emulsion as being a microemulsion or a nanoemulsion. The present investigation revealed that the developed formulations had a droplet size of between 61 ± 1.5 and 138 ± 3.1 nm (see Table 2), with an acceptable polydispersity index ranging from 0.09 to 0.40, confirming the acceptable homogeneity and favorable size distribution of the developed formulations.

A quadratic model of polynomial analysis was employed to scrutinize the collected data on droplet size. The chosen design showed the employed model’s competence in exploring the influence of the amount of Gr oil (A), amount of PV (B), and HLB of the surfactant mixture (C) on the developed emulsions’ droplet size. The advocated mathematical model had an adjusted R^2^ value of 0.9729 and predicted R^2^ value of 0.9445, which were closely related, as shown in Table 3. The ANOVA analysis of the data provided the following equation.

(2) $$\mathrm{Globule}\;\mathrm{size}=+78.26+14.87A+1.29B+0.1909C+3.04\mathrm{AB}-0.1560\mathrm{AC}-1.51\mathrm{BC}+0.5087A^{2}-2.68B^{2}+\;35.64C^{2}$$

As perceived, the amount of Gr oil (factor A) had a significantly agonistic action on the droplet size at a *p*-value of less than 0.0001; thus, any increase in the amount of Gr oil would eventually increase the droplet size. Increasing the amount of Gr oil might have provided more space in which the PV could be housed within the nanoemulsion, giving droplets a larger diameter. Further, the increase in the amount of Gr oil would be accompanied with a corresponding decrease in the amount of cosurfactant used and its capacity to downsize a nanoemulsion’s droplets, leading to the formation of larger droplets, as similar findings had previously reported [36]. On the other hand, the amount of PV and HLB value of the surfactant mixture exerted an insignificant effect on the droplet size.

**Figure 1:**
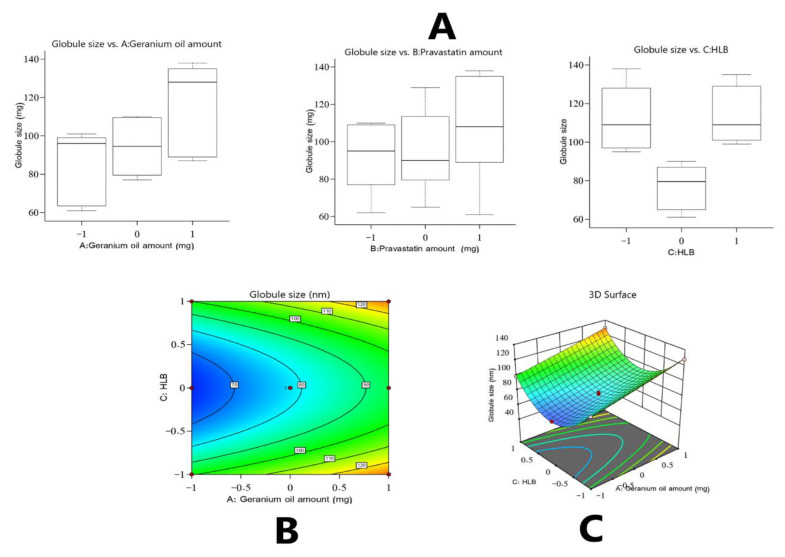
Main effect diagram (**A**), contour plot (**B**), and three-dimensional (3D) surface plot (**C**) showing the effects of different independent variables on the droplet size (Y_1_) of different Gr-PV-NE formulations.

Based on the above equation and Figure 1, which show the effect of the studied factors on the droplet size, an interesting result was detected: There was a significant impact exerted by the quadratic term of factor C (i.e., C^2^) on the droplet size of the developed formulations. As shown in Figure 1A, such an observation implies that the peripheral levels of factor C had a positive effect on the droplet size, whereas the middle level exerted a negative effect, yielding droplets with a smaller diameter. Such phenomena could be due to the fact that each lipid material must have a particular HLB value to lower the nanoemulsion droplet size, as reported by other researchers [43,44]. Therefore, it might be deduced that the best HLB value for the Gr oil used in the formulations should be the middle value among those used in the proposed design.

### 3.2. Assessment of Wound Healing

The healing of open wounds is a complicated dynamic chain of events that encompasses some sequential and overlapping processes, including hemostasis, inflammation, epithelialization, cell proliferation, revascularization, and collagen development [45]. In the present investigation, the healing impact of the fabricated formulations on burn wounds was determined by measuring the wound diameter. The diameters fluctuated between 2.5 ± 0.30 and 10 ± 1.2 mm (see Table 2) and followed a quadratic model of polynomial analysis. The chosen experimental design employed the model’s adequacy to observe the impact of independent variables on the diameter of burn wounds (Y_2_). The statistical model had an adjusted R^2^ value of 0.9817, which was in line with an expected R^2^ value of 0.9642, as shown in Table 3. The ANOVA analysis of the collected data yielded the following equation.

(3) $$\mathrm{Mean}\;\mathrm{burn}\;\mathrm{wound}\;\mathrm{diameter}=+6.03-1.64A-1.51B+0.0377C+0.5102\mathrm{AB}+0.1786\mathrm{AC}+0.1548\mathrm{BC}-\;0.3194A^{2}-0.2792B^{2}+0.41108C^{2}$$

**Figure 2:**
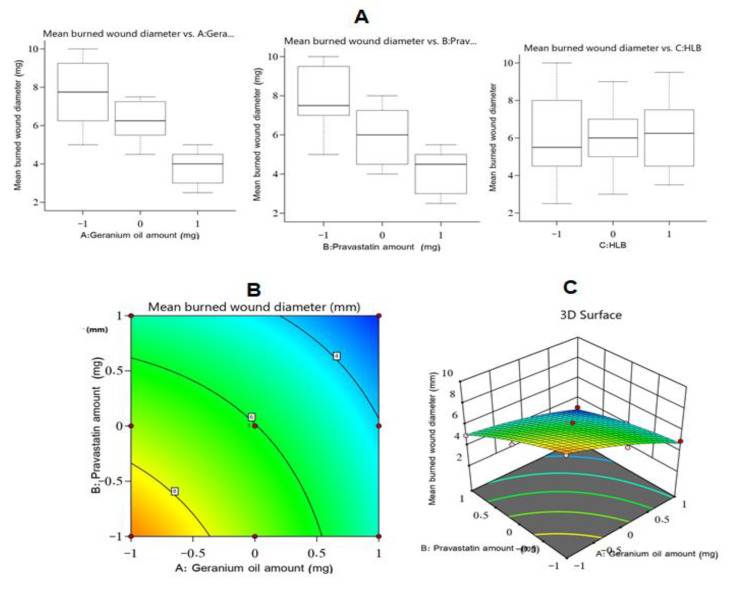
Main effect diagram (**A**), contour plot (**B**), and 3D surface plot (**C**) showing the effects of different independent variables on the mean burn wound diameter (Y_2_) obtained after the application of different Gr-PV-NE formulations.

It was noticed that there was an inverse relationship between the amounts of Gr oil and PV and response Y_2_ values at a *p*-value of less than 0.0001. The potential of the PV to decrease the burn wound size might be related to its ability, as a member of the statin class of drugs, to promote the output of the VEGF at the injury site; VEGF is a key element for developing new blood vessels [46]. Further, PV is also thought to inhibit mevalonate and farnesyl pyrophosphate production, leading to enhanced epithelialization and renovation of tissues of wounded skin [47]. Additionally, the wound healing action of Gr oil (factor A) might be due to its strong antibacterial activity against the Gram-negative bacterial strains that are mostly responsible for wound infections and that are resistant to treatments, and similar findings were reported in the literature [45]. The influence of the studied factors on the mean burn wound diameter is shown in Figure 2.

### 3.3. Optimization and Evaluation of Nanoemulsion Formulations

**Figure 3:**
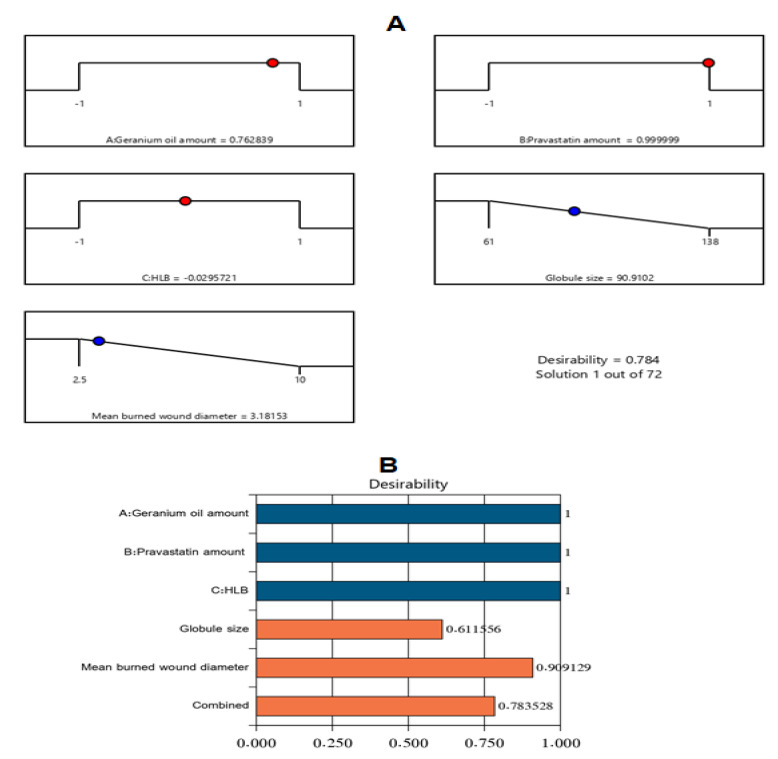
Desirability ramp and bar chart for optimization. (**A**) The desirability ramp shows the levels of independent variables and predicted values for the responses of the optimum formulation. (**B**) The bar chart shows the desirability values for the combined responses.

Following the completion of the tests described, a nanoemulsion formulation with the most appropriate specifications (i.e., the optimum formulation) was defined. Varying combinations of independent variables were suggested by the experimental design. The optimum formulation had 275 mg of Gr oil, 40 mg of PV, and a surfactant mixture with an HLB of 12 with a desirability value of 0.784. The fabricated optimal Gr-PV-NE had a droplet size of 95 ± 2.4 nm and a mean burn wound diameter of 3 ± 0.3 mm. Such results were in close accordance with the predicted values of the same responses, which were 90 nm for the droplet size and 3.18 mm for the mean burn wound diameter. Figure 3 clarifies the desirability ramp and bar chart for different levels of the studied factors and predicted dependent variables of the optimal formulation. Optimum formulation acquired an EE% of 91.3 ± 2.6% and ZP value of −17.3 ± 1.2 mV indcating the acceptable drug loading and stability of the developed optimal formulation.

#### 3.3.1. Wound Healing Action Assessment

##### Burn Wound Diameter Evaluation

**Figure 4:**
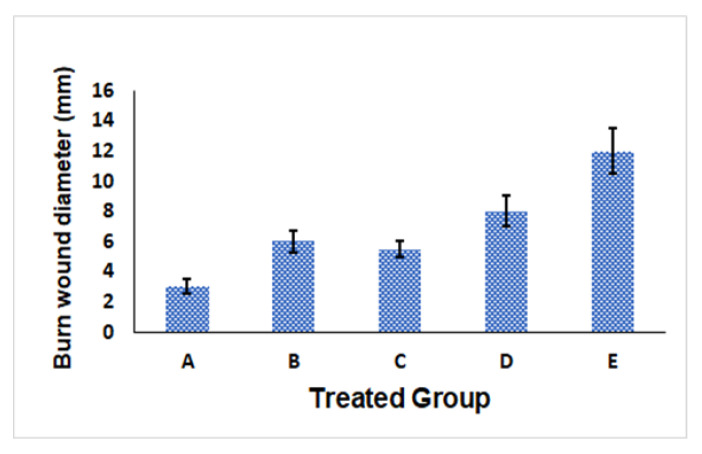
Mean burn wound diameter for different formulations: optimum Gr-PV-NE (**A**), Gr-NE (**B**), PV-NE (**C**), PV-Gr mixture (**D**), and normal saline (**E**).

**Figure 5:**
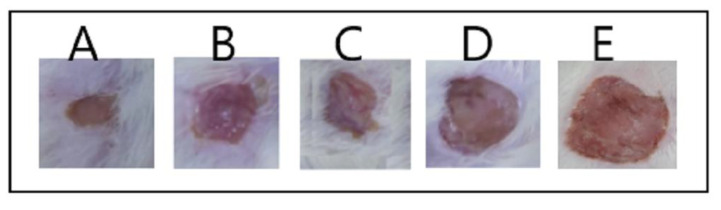
Mean burn wound diameter for different formulations: optimum Gr-PV-NE (**A**), Gr-NE (**B**), PV-NE (**C**), PV-Gr mixture (**D**), and normal saline (**E**).

As can be observed in Figure 4, the optimum Gr-PV-NE (formulation A) had the lowest mean burn wound diameter of 3 ± 0.5 mm, while the group treated with normal saline (formulation E) had the largest wound diameter, 12 ± 1.5 mm, compared with the other tested formulations. It was also noticed that the nanoemulsion containing no PV (formulation B) had a wound diameter (i.e., 6 ± 0.75 mm) greater than that of formulation A but very close to that of formulation C (i.e., 5.5 ± 0.5 mm), which was a nanoemulsion containing oleic acid instead of Gr oil. Such outcomes affirm the synergistic wound healing activity of PV and Gr oil. On the other hand, the Gr-PV mixture (formulation D) had a mean burn wound diameter of 8 ± 1 mm, which was greater than that of the optimum nanoemulsion formulation, indicating the predominant wound healing activity of the nanosized formulation compared with the mixture of Gr oil and PV. Figure 5 also illustrated the wound healing results of rat skin after 14 days of treatment The obtained rsults were found to be significant at asignificance level of 0.05 (*p*-value < 0.05)

##### IL-6 Level Evaluation

IL-6 is an abundant cell protein that helps in modulating immune system responses. The IL-6 level is usually raised by triggers such as injuries, inflammatory conditions, microbial infections, disturbances of the immune system, and malignant tumors; accordingly, IL-6 could be an advantageous marker for detecting inflammation and immune system activation [48].

**Figure 6:**
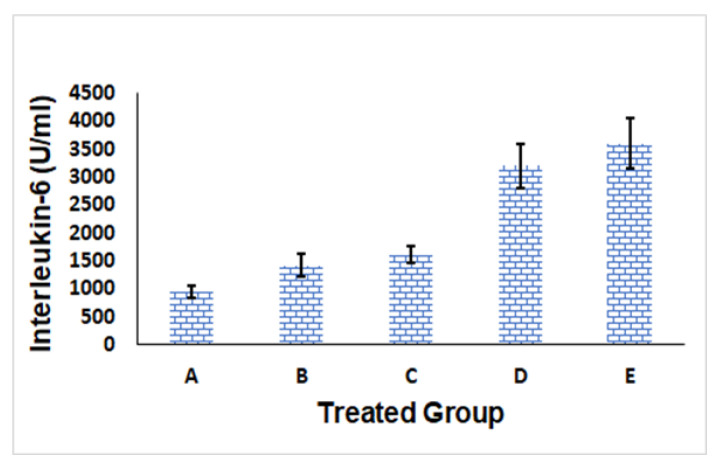
IL-6 levels for different formulations: optimum Gr-PV-NE (**A**), Gr-NE (**B**), PV-NE (**C**), PV-Gr mixture (**D**), and normal saline (**E**).

As could be seen in Figure 6, formulation A (i.e., Gr-PV-NE) had the lowest IL-6 serum level of 944 ± 100 U/mL, whereas formulation E (i.e., normal saline) had the highest level of IL-6 (3600 ± 450 U/mL); this indicated the superiority of formulation A in counteracting inflammation compared with the other tested formulations. The superior anti-inflammatory activity of the optimum nanoemulsion formulation could be due to its content of PV and Gr oil [49]. It is well known that statins can depress the output of pro-inflammatory cytokines [50] via inhibiting HMG-CoA reductase, which could invigorate the mevalonate pathway. As a result, PV might minimize the occurrence of the isoprenylation and geranylgeranylation of proteins, particularly Ras protein prenylation. The suppression of Ras diminishes the efficiency of transcription factor nuclear factor kappa B, which plays a pivotal role in many inflammatory reactions [51].

The anti-inflammatory action of Gr oil might be connected to the prohibition of some intracellular signaling pathways encompassing several inflammatory mediators’ actions. Abe et al. [52] revealed the ability of Gr oil to depress the adherence response of human neutrophils in vitro and diminish the induced neutrophil mobilization in the peritoneal cavity following the intraperitoneal administration of the oil. Several studies have explored the components that might be responsible for such bioactivity [53]. It was found that the major components of the oil, namely, geraniol, citronellol, and linalool, were proven to have anti-inflammatory influences [54,55].

Formulations B and C had Il-6 values of 1415 ± 200 U/mL and 1611 ± 150 U/mL, respectively. Although these values indicated a higher effect of formulation C, which contained PV and oleic acid instead of Gr oil in the nanoemulsion, compared with formulation B, which contained only Gr oil in the nanoemulsion, such difference was found to be insignificant using Tukey post test. Moreover, formulation D, which was composed of PV and Gr oil, had an IL-6 value of 3200 ± 400 U/mL, affirming the favorable anti-inflammatory behavior of the optimum nanoemulsion formulation (formulation A).

IL-6 serum levels of the different tested groups were examined for normal distribution characteristics using the K-S test of normality. The observed high *p*-values and low D-values for all of the formulations suggested that there was no considerable difference between the collected data and the data that were normally distributed.

The outcomes of the ANOVAs confirmed that all of the tested formulations (except for formulation D) had much higher IL-6 levels than formulation E, with a *p*-value of less than 0.01; therefore, the noticed variations between formulations could not happen by chance. The Tukey HSD post-hoc test revealed that the IL-6 serum levels of all of the formulations varied significantly from each other (*p*-value < 0.01), except that the comparisons between formulations C and B and D and E were found to be insignificant. Such findings are quite reasonable because formulation B and formulation C each contained one component (i.e., PV in the case of B and Gr oil in the case of C) that had an IL-6–lowering effect.

#### 3.3.2. Ex-Vivo Permeation Study

**Figure 7:**
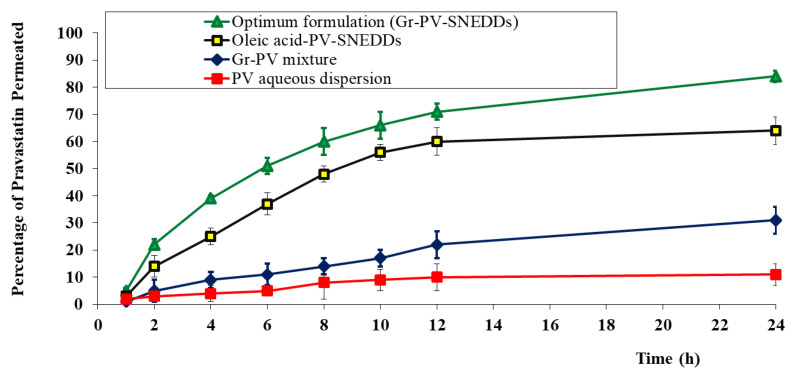
Ex-vivo permeation profiles of PV from different tested formulations.

Upon reviewing the ex-vivo permeation results presented in Table 4 and Figure 7, the following information was detected. First, the nanoemulsion formulation that contained PV and oleic acid instead of Gr oil promoted drug permeation across the skin by more than 5.8 when compared with the PV aqueous dispersion. More importantly, the optimized Gr-PV-NE encouraged skin permeation of PV by 7.6-fold and 2.7-fold in comparison with the PV aqueous dispersion and PV-Gr physical mixture, respectively. Such enhancement might be due to a synergistic effect of a nanoemulsion as a drug delivery system and the effect of Gr oil (which contains components like citronellol and geraniol, which acted as penetration enhancers) on the permeation by PV; similar results were found in the literature [44]. Nanosized emulsions are known to offer a large surface area for drug permeation, in addition to their surfactant and cosurfactant contents, which are thought to fluidize the stratum corneum layer, which is the main barrier to the permeation of drugs through the skin [56]. The obtained rsults were found to be significant at a significance level of 0.05 (*p*-value < 0.05).

#### 3.3.3. Antibacterial Activity Assessment

Table 4 shows the collected antibacterial activity data, which revealed the following. The optimum Gr-PV-NE had the largest growth inhibition zone against *S. aureus* (20 ± 1.8 mm) compared with the other tested formulations. The antimicrobial activity of the optimized formulation appeared to be mainly due to its Gr oil content, which is known to have significant antibacterial activity against *S. aureus* due to its components of citronellol, geraniol, linalool, isomenthone, nerol, and citronellyl formate [57]. Such components exert their antimicrobial effects by interacting with the bacterial cell membrane, increasing its fluidity and leading to the leakage of cell components [57]. Such results were further confirmed by comparing the growth inhibition zones for formulations that contained Gr oil with those that contained no Gr oil. Results indicated that the formulations that contained Gr oil enhanced the antimicrobial activity by four times compared with those that did not contain Gr oil. The NE containing Gr oil had 1.6 times the antimicrobial activity as the physical mixture; in the N, the Gr oil present in the nanosized dispersion enhanced the permeation of the formulation across the microbial membrane and potentiated its action. The obtained rsults were found to be significant at asignificance level of 0.05 (*p*-value < 0.05).

## 4. Conclusions

The present investigation utilized the BBD for the characterization and optimization of a Gr oil–based nanoemulsion loaded with PV for the transdermal management of burn wounds. The fabricated nanoemulsions had a reasonable droplet size of 61 to 138 nm. The experimental design confirmed the substantial synergistic effect of the Gr oil and PV for burn wound healing. Such a blend increased the management of the wound healing and the anti-inflammatory character and antibacterial effects of each of the constituents. The optimal formulation had up to a 4-fold decrease in the mean burn wound diameter, a 3.81-fold lowering of the IL-6 serum level, a 4-fold increase in the inhibition zone against *S. aureus*, and a 7.6-fold increase in PV permeation upon comparison with different formulations. It was conclusively seen that the obtained nanoemulsions that contained a combination of Gr oil and PV could be considered a promising paradigm for handling burn wounds.

## Figures and Tables

**Table 1:** Independent variables and their levels, along with dependent variables and their constraints, in the BBD of the nanoemulsion formulations.

Independent Variables	Levels		
	−1	0	1
A = Geranium oil amount (mg)	100	200	300
B = Pravastatin amount (mg)	10	20	40
C = HLB of surfactant mixture	11	12	13
Dependent Variables	Goal		
Y_1_ = Globule size (nm)	Minimize		
Y_2_ = Mean burned wound diameter (mm)	Minimize		

**Table 2:** BBD and responses of Gr-PV-NEs.

	A	B	C	Y_1_	Y_2_	
Run	Geranium Oil Amount (mg)	Pravastatin Amount (mg)	HLB	Droplet Size (nm)	Mean Burned Wound Diameter (mm)	Polydispersity Index
1	200	10	13	110 ± 2.0	7.5 ± 0.30	0.09 ± 0.02
2	300	20	11	128 ± 4.5	4.5 ± 0.18	0.11 ± 0.03
3	100	20	11	99 ± 7.0	8.0 ± 0.90	0.15 ± 0.03
4	100	10	12	62 ± 1.5	9.0 ± 1.10	0.21 ± 0.04
5	300	20	13	129 ± 3.2	4.5 ± 0.62	0.13 ± 0.02
6	100	40	12	61 ± 1.5	5.0 ± 0.51	0.32 ± 0.04
7	200	20	12	81 ± 5.0	6.5 ± 0.25	0.28 ± 0.05
8	200	20	12	80 ± 4.5	6.0 ± 0.18	0.19 ± 0.04
9	300	40	12	89 ± 3.5	3.0 ± 0.09	0.22 ± 0.05
10	200	10	11	109 ± 4.0	7.5 ± 0.64	0.38 ± 0.06
11	200	40	13	108 ± 8.0	5.0 ± 0.33	0.40 ± 0.05
12	200	40	11	110 ± 2.5	4.5 ± 0.21	0.10 ± 0.02
13	200	20	12	79 ± 1.9	6.0 ± 0.30	0.35 ± 0.02
14	100	20	13	99 ± 4.0	7.5 ± 0.42	0.30 ± 0.04
15	300	10	12	87 ± 3.5	5.0 ± 0.17	0.29 ± 0.05
16	100	10	11	95 ± 2.9	10 ± 1.20	0.26 ± 0.05
17	300	40	13	135 ± 5.5	3.5 ± 0.09	0.18 ± 0.04
18	100	10	13	101 ± 2.1	9.5 ± 0.50	0.32 ± 0.03
19	100	40	11	97 ± 6.1	5.5 ± 0.11	0.27 ± 0.05
20	300	20	12	90 ± 1.5	4.0 ± 0.30	0.19 ± 0.06
21	300	40	11	138 ± 3.1	2.5 ± 0.30	0.11 ± 0.03
22	100	20	12	65 ± 2.0	7.0 ± 1.90	0.25 ± 0.05
23	200	10	12	77 ± 1.8	7.0 ± 0.21	0.39 ± 0.06

**Table 3:** Regression analysis results for Y_1_ and Y_2_ responses.

Dependent Variables	R^2^	Adjusted R^2^	Predicted R^2^	F-Value	*p*-Value	Adequate Precision
Y_1_	0.9840	0.9729	0.9445	88.79	0.0001	30.3760
Y_2_	0.9892	0.9817	0.9642	132.17	0.0001	38.5081

**Table 4:** Inhibition zones against *S. aureus* and percentage PV permeated for varying formulations.

Run	A: Geranium Oil Amount	B: Pravastatin Amount	C: HLB Value	Pravastatin Permeated %	Inhibition Zone against *S. aureus* (mm)
Optimum formulation (Gr-PV-NE)	275 mg	40	12	84% ± 3.1	20.0 ± 1.8
Gr-NE	275 mg	0	12	0	19.0 ± 1.4
Oleic acid-PV-NE	0	40	12	64% ± 2.4	6.00 ± 0.8
Gr-PV mixture	275 mg	40	0	31% ± 1.2	12.0 ± 1.1
PV aqueous dispersion	0	40	0	11% ± 0.6	4.50 ± 0.3
Plain NE	0	0	12	0	5.00 ± 0.5

## Data Availability

All data available are reported in the article.
